# Practice of parenteral nutrition in hospitalized adult patients in Korea: A retrospective multicenter cross-sectional study

**DOI:** 10.1371/journal.pone.0230922

**Published:** 2020-04-01

**Authors:** Hyo Jung Park, Jung Tae Kim, Jee Eun Chung, Jin A. Yang, Hye Jung Bae, Ye Won Sung, Ji Eun Park, Sun Hwa Kim, Ji Yoon Cho, Kyung Mi Jung, Hee Kyung Bae

**Affiliations:** 1 Department of Pharmaceutical Services, Samsung Medical Center, Seoul, Republic of Korea; 2 Department of Pharmacy, Kyung Hee University Hospital at Kangdong, Seoul, Republic of Korea; 3 College of Pharmacy, Hanyang University, Ansan, Gyeonggi-do, Republic of Korea; 4 Department of Pharmacy, Asan Medical Center, Seoul, Republic of Korea; 5 Department of Pharmacy, Seoul National University Hospital, Seoul, Republic of Korea; 6 Department of Pharmacy, Chungnam National University Hospital, Daejeon, Republic of Korea; 7 Department of Pharmacy, Severance Hospital, Seoul, Republic of Korea; 8 Department of Pharmacy, Chung Ang University Hospital, Seoul, Republic of Korea; 9 Department of Pharmacy, Daegu Fatima Hospital, Daegu, Republic of Korea; 10 Department of Pharmacy, Ulsan University Hospital, Ulsan, Republic of Korea; Wayne State University, UNITED STATES

## Abstract

There have been no studies on the characteristics of parenteral nutrition (PN) supply for adult inpatients in South Korea. The aim of this retrospective multicenter cross sectional study was to investigate the current practice and characteristics of PN support in hospitalized adult patients in South Korea for the first time. This study was conducted retrospectively for the adult patients who were hospitalized and received PN in nine hospitals on August 1st, 2017 to October 30^th^, 2017. We evaluated the type of PN formulation, PN administration period, administration route, calories supplied, amount of protein supplied, and laboratory results. Among the 11,580 inpatient admissions on that day, 1,439 patients received PN (12.4%). The majority of enrolled patients (96.5%) used the commercial PN, of which 86.2% were multi-chamber. 71.2% of them received PN peripherally. The average in hospital PN duration was 17.8 ± 52.6 days. Patients received only 65.4 ± 25.4% calories of their target calories. The in-hospital mortality of enrolled patients was 22%. In South Korea, commercial PN was usually administered to hospitalized adult patients and in-hospital mortality in adult patients using PN was higher in South Korea compared to other countries. This study provides the characteristics and the PN support status of hospitalized adult patients receiving PN in South Korea.

## Introduction

Malnutrition is associated with longer hospital stays, greater in-hospital mortality, and readmissions [1–3]. To prevent negative consequences due to malnutrition, the American Society for Parenteral and Enteral Nutrition (ASPEN)/Society of Critical Care Medicine (SCCM) and the European Society for Clinical Nutrition and Metabolism (ESPEN) [4, 5] recommend providing parenteral nutrition (PN) to the patients if they are severely malnourished when they are hospitalized or sufficient calories are not allowed for an extended time by oral or enteral feeding [6–9]. Many hospitals in South Korea supply PN in accordance with ASPEN and ESPEN guidelines. In South Korea, the prevalence of malnutrition in the hospitalized adult patients reportedly ranges from 22 to 61% [10, 11]. However, the clinical practices and characteristics of current PN formulations for inpatients who receive PN are not known.

The aim of the study was to investigate the characteristics and the PN support status of hospitalized adult patients receiving PN in South Korea, in order to provide basic data for future study.

## Methods

The clinical nutrition committee of the Korean Society of Health-System Pharmacists (KSHP) and clinical research groups of the Korean Society for Parenteral and Enteral Nutrition (KSPEN) recruited nine hospitals to participate in this study from among KSHP/KSPEN member hospitals. A total of nine hospitals composed of seven tertiary hospitals and two general hospitals were finally enrolled in this study. Study participants were hospitalized and received PN on August 1^st^, 2017. The study ended on October 30^th^, 2017. Patients who were pregnant or less than 19 years old, considered as the minors in Korean law were excluded. Patients were divided into four groups according to the PN administration period: four or less days, four to seven days, eight to fourteen days and more than fourteen days.

We retrospectively investigated the following characteristics by reviewing the electronic medical records (EMR) in eight hospitals: age, sex, height, weight, department of admission, major diagnosis at the time of admission, and laboratory data. Samsung Medical Center (SMC) used a database called clinical Data Warehouse, which is designed to search and retrieve de-identified patient information from electronic medical records to protect the patient’s personal information. We evaluated the type of PN formulation, PN administration period, administration route, the calories supplied and amount of protein supplied, whether to refer to the nutrition support team (NST), whether to refer to nutrition support team, reports of adverse drug reactions on PN-related complications, and laboratory results such as the electrolytes, liver function tests (LFTs), blood urea nitrogen (BUN), serum creatinine (Scr), and trace elements. All data were fully de-identified before access. All participating hospitals received approval from IRB. The Institutional Review Board of Samsung Medical Center approved this study (approval number: 2017-10-143) and the IRB waived the requirement for informed consent.

PN duration was calculated as the dates between the PN start and end dates. We calculated the duration based on October 30^th^ as the end date, even though the PN intake continued beyond October 30^th^. We divided the patients into groups based on their surgery history. The surgery group was for patients who had surgery within 15 days before or after the data collection date. The non-surgery group included patients who did not have surgery during that time period. We analyzed the data by the period of PN interruption to death.

The actual amount of energy supplied was compared with each patients’ target calories and the achievement rate was obtained. We analyzed abnormal laboratory levels to check the occurrence of PN-induced complications. Biochemical laboratory data were collected within one week before and after August 1^st^, 2017. For trace elements, we collected the laboratory data between July 1^st^ and August 1^st^. Because this is a retrospective study, there is a possibility that some of the medical record may be missing some PN-related complications. We checked for the reports of adverse drug reactions on PN-related complications in registered patients.

Data are presented as mean and standard deviation for continuous variables and as numbers (percentages) for categorical variables. Data were compared using the chi-square test or Fisher’s exact test for categorical variables and Student’s t-test or Mann-Whitney test for continuous variables, as appropriate. IBM SPSS Statistics version 25, SAS version 9.4 (SAS Institute, Cary, NC) was used for analysis.

## Results

Among the 11,580 inpatient admissions on August 1^st^, 2017, 1,439 patients received PN (12.4%). The basic characteristics of the participating hospitals are listed in Table 1.

**Table 1:** Characteristics of participating hospitals (*n* = 9).

Name of hospital	Type of hospital	Number of beds for inpatients	Number of patients receiving PN	Number of pharmacists in nutrition support team	Method to calculate target calories for adult patients	Type of hospital- compounded PN
K.D.	General	623	78	2	Equations, Weight based calories	2-in-1
D.P.	General	710	23	1	Equations, Indirect Calorimetry	3-in-1
S.S.	Tertiary	1,972	146	10	Equations, Weight based calories	2-in-1
S.N.	Tertiary	1,786	141	11	Equations, Weight based calories	3-in-1, 2-in-1
S.A.	Tertiary	2,700	320	3	Weight based calories	2-in-1
Y.S.	Tertiary	2,400	343	5	Equations, Weight based calories	2-in-1
U.D.	Tertiary	986	114	2	Equations, Weight based calories	No hospital- compounded PN
C.A.	Tertiary	820	60	2	Weight based calories	No hospital- compounded PN
C.N.	Tertiary	1,387	214	1	Equations, Weight based calories	Individual nutrients supply

2-in-1: dextrose-amino acid-micronutrients formulation, 3-in-1: dextrose-amino acid- fat emulsion-micronutrients formulation, PN: parenteral nutrition

Demographics for the enrolled patients that received PN are described in Table 2. Of those on PN, the average age was 62.5 ± 15.4 years, the average height was 162.0 ± 9.3 cm (*n* = 1,379), the average weight was 57.4 ± 12.5 kg (*n* = 1,420). More male patients (56.6%) received PN. The department of General Surgery represented the largest total of hospitalized patients, followed by Hematology-oncology, and Gastroenterology. Among the patients who received PN, the Hematology-oncology department had the highest percentage at 27.2%, followed by General Surgery and Gastroenterology. Most of the patients were in non-Intensive Care Unit (ICU) settings, and only 8.7% were in the ICU.

**Table 2:** Demographics for the enrolled patients.

	*n* = 1439
Sex, *n*(%)	
- Male	814 (56.6)
- Female	625 (43.4)
Age, years	62.5±15.4
Height, cm	162.0±9.3
Weight, kg	57.4±12.5
Body mass index, kg/m^2^	21.8±4.0
BMI, *n*(%)	
- ≥18	277 (19)
- 18–24.9	829 (58)
- ≥25	264 (18)
Primary diagnosis of the patient according to Korean Standard Classification of Diseases, *n*(%)	
- Neoplasms	761 (53)
- Diseases of the digestive system	186 (13)
- Diseases of the respiratory system	87 (6)
- Diseases of the circulatory system	74 (5)
- Diseases of the blood and blood-forming organs and certain disorders involving the immune mechanism	54 (4)
- Others	277 (19)
Department, *n*(%)	
- Hematology-oncology	392 (27)
- Surgery	282 (20)
- Gastroenterology	271 (19)
- Pulmonology	100 (7)
- Neurosurgery	66 (5)
- Others	328 (23)
PN administration routes, *n* (%)	
- Central line	415 (28.8)
- Peripheral line	1,024 (71.2)
Cancer	
- Yes	761 (53)
- No	678 (47)
Surgery	
- Yes	410 (28)
- No	1,029 (62)
Mortality, *n*(%)	317/1,121 (22)
- Cancer	210/317 (66)
- Surgery	59/317 (19)
- ICU	51/317 (16)
Ward, *n* (%)	
General ward	1,314 (91.3)
- ICU	125 (8.7)

BMI: body mass index, ICU: intensive care unit

For the ratio of the patients who received PN out of the total population for each department, Family Medicine had the largest percentage at 40% (n = 10 out of 24), followed by Emergency Medicine at 27.3% (n = 12 out of 44). The portion of the Dentistry (n = 5 out of 25) and Allergy departments was also 20% (n = 1 out of 5).

Because we analyzed patients who had PN on August 1^st^ 2017, most of them started PN prior to that date. The average PN duration was 17.8 ± 52.6 days, ranging from one day to 1,780 days. Each group of four or less days, four to seven days, eight to fourteen days and more than fourteen days accounted for 20.9%, 22.9%, 23.8% and 32.4% of all patients, respectively. 84.4% of the patients received the PN within a four-week period.

The number of patients in the surgery group was 410, which was 28.5% of enrolled patients. The surgery group had a shorter PN period than the non-surgery group (p<0.001). The surgery group had an average PN period of 10.9 ± 14.3 days, while the non-surgery group has an average PN period of 20.5 ± 61.3 days.

### Type of PN formula

The majority (96.5%) of enrolled patients used commercial PN, and only 3.5% used hospital-compounded PN. 86.2% of the commercial PN were multi-chamber bags composed of dextrose, amino acids, lipid and micronutrients. There were a few cases in which fat emulsion and amino acids were provided additionally to commercial PN. More patients received PN from the peripheral line (71.2%) than from the central line.

When being administered hospital-compounded formulas (n = 50, 3.5%), 62.7% of the patients were given 2-in-1 PN, including dextrose and amino acids and the others received 2-in-1 PN and fat emulsion together.

Among patients who were supplied with intravenous lipids, 78.8% were administered intravenous lipids, which were from fat emulsion containing fish oil, i.e., from soybean, medium-chain triglycerides, olive oil, and fish oil. The fat emulsion-based olive oil and soybean was supplied to 16.0% of the patients. The soybean oil with medium-chain fat emulsion and soybean oil alone were administered to 3.8% and 1.4% of patients, respectively.

### Nutrient supplied

On average, patients were provided with 1,030.9 ± 345.3 kcal/day (19.6 ± 7.6 kcal/kg/day) and 47.5 ± 19.2 grams of amino acids (0.9 ± 0.4 g/kg/day). Overall, the patients received only 65.4 ± 25.4% calories of their target calories. The supplied calories ranged from 15.4% to 191.2%.

Only 51.8% patients were provided a multivitamin, and 74.8% of patients were not provided with the trace elements. About 20% of the patients received trace element complex products, and a few patients received a single trace supplement, such as zinc or selenium.

### Laboratory monitoring and complications

Table 3 shows the percentage of patients with normal values and means of all the patients who participated in the study.

**Table 3:** Biochemical laboratory results.

	% (No of patients with normal values / No of patients who underwent the test)	Mean ± SD	Min	Max
Sodium (mEq/L)	68.3 (955/1,398)	136.8±5.2	107	161
Potassium (mEq/L)	80.0 (1,119/1,398)	4.0±0.6	2.3	7.4
Phosphorus (mg/dL)	75.7 (970/1,282)	3.2±1.0	0.8	10.3
Magnesium (mg/dL)	81.9 (370/452)	2.0±0.4	0.68	3.8
Calcium (mg/dL)	40.0 (534/1,336)	8.4±0.7	6.2	16.5
Ionized Ca (mg/dL)	35.1 (184/524)	4.6±0.4	3.44	6.8
Albumin (g/dL)	27.7 (384/1,386)	3.0±0.7	0.9	5.4
Total bilirubin (mg/dL)	79.0 (1,082/1,369)	1.5±3.5	0.1	45.3
Direct bilirubin (mg/dL)	59.3 (308/519)	1.8±4.3	0	39.3
Glucose (mg/dL)	85.2 (1,097/1,288)	137.0±52.1	48	592
AST (U/L)	76.2 (1,055/1,385)	49.9±172.5	5	4078
ALT (U/L)	81.2(1,124/1,385)	36.8±86.9	1	1721
ALP (U/L)	65.6 (889/1,357)	134.8±165.0	24	3198
BUN (mg/dL)	63.3 (879/1,389)	19.7±15.4	2	141
SCr (mg/dL)	38.9 (541/1,391)	0.9±1.0	0.17	11.74
Zinc (mcg/dL)	82.4 (14/17)	80.5±19.0	52.9	120.9
Selenium (mcg/L)	4 (4/10)	72.9±20.2	45.7	110.1
Copper (mcg/dL)	70.6 (12/17)	94.3±33.0	36.2	170.2
Manganese (mcg/L)	50 (1/2)	0.7±0.5	0.37	1.03
Chromium (mcg/L)	100 (1/1)	2.5	2.5	2.5

Overall, electrolytes, LFTs, BUN, and Scr were monitored frequently in 97.1%, 96.2%, and 96.7% of all patients, respectively. However, magnesium and direct bilirubin monitoring were relatively infrequent, in 31.9% and 36.0% of the enrolled patients, respectively. Additionally, trace elements such as zinc, selenium, manganese, copper, and chrome were rarely collected. There were several cases in which the trace element value was lower than the normal value, and there was one case in which the copper value was higher than the normal value.

We collected the usage of insulin with PN to indirectly monitor the prevalence of hyperglycemia. 14.5% of the patients used regular insulin (RI), and patients with RI had higher blood glucose levels in PN than patients without RI administration (*p* = 0.001). However, hypoglycemia was less than 1%. There were no report of adverse drug reactions on PN-related complications.

Values of albumin, sodium, blood glucose levels, alkaline phosphatase, and Scr statistically significantly increased in a linear pattern in the three groups of BMI (BMI less than 18 kg/m^2^ group, BMI between 18 to 24.9 kg/m^2^ group and BMI more than and equal to 25 kg/m^2^ group) (Table 4).

**Table 4:** Comparison of number of patients with normal laboratory values by BMI group.

	BMI, <18 kg/m^2^	BMI, 18–24.9 kg/m^2^	BMI ≥25 kg/m^2^	*p value*
Sodium (mEq/L)	149/266 (56)	502/807 (62)	176/256 (69)	0.011
Potassium (mEq/L)	215/266 (81)	660/807 (82)	209/256 (82)	0.94
Phosphorus (mg/dL)	181/248 (73)	543/736 (74)	180/236 (76)	0.677
Magnesium (mg/dL)	72/87 (83)	207/239 (87)	64/74 (86)	0.665
Calcium (mg/dL)	114/261 (44)	302/765 (39)	99/249 (40)	0.478
Ionized Ca (mg/dL)	65/96 (68)	163/279 (58)	63/109 (58)	0.236
Albumin (g/dL)	59/267 (22)	220/798 (28)	85/254 (33)	0.015
Total bilirubin (mg/dL)	226/267 (85)	653/786 (83)	198/249 (80)	0.28
Direct bilirubin (mg/dL)	54/102 (53)	173/301 (57)	57/98 (58)	0.689
Glucose (mg/dL)	221/246 (90)	628/745 (84)	196/238 (82)	0.047
AST (U/L)	185/268 (69)	539/796 (68)	169/254 (67)	0.83
ALT (U/L)	213/268 (79)	614/796 (77)	181/254 (71)	0.068
ALP (U/L)	167/261 (64)	567/780 (73)	197/251 (78)	0.001
BUN (mg/dL)	200/268 (75)	594/800 (74)	195/253 (77)	0.662
SCr (mg/dL)	129/269 (48)	314/800 (39)	80/254 (31)	0.001

Number of patients with normal values / Number of patients who underwent the test (%)

### Clinical outcomes

Hospital mortality was 22%. There were 761 patients (58%), 410 patients (28%), and 125 patients (8.7%) who were hospitalized for cancer, surgery and intensive care, respectively. Mortality was 210 (53%), 59 (28%), and 51(41%), respectively. 27.8% of patients received PN on the day of death, and more than 50% of patients received PN within three days of death.

### Nutrition support team effect

Among all the patients who received PN, about 70% received PN without nutrition support team (NST) consultation, and only 30% were consulted by NST.

Patients in the NST consulting group had a higher percentage of goal calories (75.7 vs. 61.5%, *p* < 0.001), a higher supply of amino acids (1.04 vs. 0.79 g/kg, *p* < 0.001), a longer PN supply (32.9 vs. 11.5 days, *p* <0.001), a longer length of hospital stay (66.0 vs. 24.6, *p* < 0.001), and a higher mortality rate (31.2 vs. 18.3%, *p* < 0.001) than patients without NST consulting.

## Discussion

Data from the Premier Perspective database, the largest inpatient clinical database in the United States, were used to evaluate hospital-based PN practices [12]. Data gathered between January 2005 and December 2007 included a total of 68,984 adults receiving PN, which was 0.77% of all adult inpatients. Among the adult PN population, 38.9% were diagnosed with a malignancy.

In our multicenter cross-sectional study in Korea, 12% of the hospitalized adult patients had parenteral nutrition and 52.9% of the PN population was diagnosed as cancer. The percentage of the PN population from the hospitalized adult patients and cancer patients in Korea with PN were 15.6 times and 1.36 times higher, respectively, when compared with the data from the Premier Perspective study. Moreover, hospital mortality of 22% and PN administration of eighteen days in the study were much higher than the 30-day mortality rate of 8% and median PN days of seven days in the study of the Northern Nutrition Network [13].

Most of the enrolled patients used the commercial peripheral PN with three-chamber bags in this study. Wajdy A.S [14] reported 39% of the PN population used commercial PN. Monica L. P [15] reported that almost 40% of subjects received PN via a peripheral catheter, for a duration greater than two weeks.

In South Korea, hospital mortality in adult patients receiving PN was higher, and commercial PN was administered to hospitalized adult patients more often than in other countries. This may be because the Korean National Health Insurance System might be different from other countries [16]. Cancer patients and hospitalized patients are more likely to receive PN treatment than patients in other countries because of the support from health insurance and relatively low cost of hospitalization. Especially, the financial burden for patients with rare and serious diseases like cancer has been substantially reduced, with patients currently paying only 5–10% of health care costs for co-payments [17].

Also, it is costly to prepare individualized PN for each hospital where there are no IV pharmacy services outside the hospital unlike foreign countries [18, 19]. Further studies are necessary to assess appropriate use of commercial peripheral PN and to find out why commercial peripheral PN is used so heavily.

Through this study, we have found that PN with three-chamber bags containing soybean, medium-chain triglycerides, olive oil, and fish oil are frequently used in South Korea. In particular, research on the use of these oils is also needed in the future.

In this study, one-half of the patients did not receive any vitamins and three quarters of those received no trace elements. Moreover, laboratory follow-up for these micronutrients was rarely monitored. This detail for administering trace elements might be easy to overlook, but still there are many hospitals that cannot test for trace elements. There may be a lack of interest and understanding of micronutrients among prescribing doctors and pharmacists who review orders. In addition, the recommended monitoring schedule is unclear. The Korean guidelines for trace element supply and laboratory follow-up should be prepared to educate healthcare providers about nutrition therapy due to the risk of micronutrient deficiency.

In the departments of family medicine, emergency medicine, dentistry and allergy, the percentage of patients who received PN out of the total population for each department was relatively high. Even though they do not have many in-patients, those departments have a high PN percentage. Education for healthcare providers and regular follow-up for appropriate PN use will be needed in these departments even with low PN usage.

Patients received only 65.4 ± 25.4% calories of their target calories. The amount of calories supplied through PN was relatively small compared to the target calories, although the amount of oral intake or enteral feeding was not collected in this retrospective study, probably because 70% of the patients were given peripheral commercial PN containing low calories to supplied IV fluid due to limited osmotic pressure.

Hypoalbuminemia was seen in 70% of patients, which is quite significant. Also, hyponatremia, hypokalemia, and hypophosphatemia were more than 10% of patients. High-ionized calcium was also 63% of patients, and high bilirubin or LFT results were more than 20% of patients. It seems that commercial PN does not fit the nutrient requirements for some inpatients.

Hypoalbuminemia and electrolyte imbalance may be appeared by malnutrition. However nutritional status was not collected in this study because each hospital have different nutrition screening system and changes of weight loss and food intake was not reported. In groups with lower BMI, the rates of hypoalbuminemia, hyponatremia increased. This indirectly suggests that hypoalbuminemia and hyponatremia may present as part of malnutrition.

In the PN patients whose PN was managed by a NST, the calories and protein supply was higher than that for the non-NST-consulted group. Active NST consultation should be done to ensure adequate nutrition supply [20]. However, NST consultation showed that that length of stay and mortality could not be improved, probably due to the greater severity of the disease in the patients who were referred to the NST.

There are several limitations in this study. First, data were collected retrospectively and there were difficulties in evaluating the effects or side effects of PN administration. Second, each hospital had a different nutrition assessment tool. We could not confirm the differences in PN supply according to nutrition status. BMI and albumin levels were investigated, but there was limitation to represent nutritional status. Third, there may be a difference between the prescribed PN dose and the actual dose administered on a daily basis, and oral ingestion without a physician’s order was also impossible to collect. Fourth, the presence or severity of the infection was not collected. Many of the biochemical measures (trace elements, albumin, etc.) were significantly affected by the presence of inflammation and the presence of inflammation will confound the results obtained [21, 22].

## Conclusions

This study provides the characteristics and the PN support status of hospitalized adult patients receiving PN in South Korea. We found out that 12.4% of hospitalized patients received nutritional support from PN in South Korea.

In contrast to the survey results in the U.S., commercial PN was used more frequently in South Korea and three-in-one PN was typically used. Normally, PN duration was less than two weeks, and this is in line with the high use of peripheral PN. Vitamins or trace elements are not provided to many patients. The results of cross-sectional studies on PN use in Korea will provide basic data for future research.
